# Acrodermatitis Enteropathica: A Case Report Involving a Delayed Diagnosis

**DOI:** 10.7759/cureus.71134

**Published:** 2024-10-09

**Authors:** Ethar A Alsulami, Manar S Alghamdi, Abdulrahman E Alraddadi, Ascia K Alabbasi, Lama Z Allehaibi, Wafi Al Hawsawi, Khalid Al Hawsawi

**Affiliations:** 1 College of Medicine, Umm Al Qura University, Makkah, SAU; 2 College of Medicine, King Saud Bin Abdulaziz University for Health Sciences, Jeddah, SAU; 3 Department of Dermatology, King Abdulaziz Hospital, Makkah, SAU

**Keywords:** acrodermatitis enteropathica, annular configuration, subacute cutaneous lupus erythematosus (scle), tinea corporis, zinc deficiency

## Abstract

Acrodermatitis enteropathica (AE) is a hereditary autosomal recessive disorder caused by a defect in zinc metabolism, leading to a zinc deficiency. We report a case of a two-year-old female infant who presented with psoriasiform-eczematous skin lesions with acral distribution in all four extremities one month after weaning from breast milk at five months old. The patient had no history of diarrhea or hair loss and had been misdiagnosed with tinea corporis and treated with topical and systemic antifungals for a year without benefit. She had also been misdiagnosed as having psoriasis and treated with corticosteroid-calcipotriene for several months without benefit. A skin examination revealed multiple well-defined patches of scaly erythematous plaque on all four extremities, and some of the lesions displayed an annular configuration. The laboratory investigation revealed a low serum zinc level (49.10 ug/dL; normal range: 60-110 ug/dL). Based on the clinicopathological findings, the patient was diagnosed with AE. She was started on 13 mg/kg of zinc sulfate syrup divided into two doses. All skin lesions completely disappeared after two weeks of zinc treatment.

## Introduction

Acrodermatitis enteropathica (AE) refers to a specific form of zinc deficiency; it is the hereditary form of zinc deficiency [1]. The acquired form of zinc deficiency is called acquired zinc deficiency [2]. Acquired zinc deficiency can result from several causes. In the pediatric age group, it can result from low birth weight, prematurity, cystic fibrosis, and transient neonatal zinc deficiency due to low zinc in breast milk [3]. The causes in both pediatric age groups and adults include HIV disease, vegan diets, anorexia nervosa, Middle Eastern diets rich in mineral-binding phytate, sickle cell disease, absorptive disorders (e.g., Crohn’s and celiac disease), post-surgical conditions (e.g., intestinal bypass), receiving parenteral nutrition, severe liver disease, nephrotic syndrome, and uremia. Pregnancy and alcoholism are the other major causes of acquired zinc deficiency. The clinical presentation of AE includes psoriasiform or eczematous skin lesions in acral and periorificial distributions, along with oral lesions, alopecia, delayed wound healing, nail changes, and systemic manifestations (e.g., diarrhea, irritability, lethargy, anorexia, growth retardation, anemia, ophthalmological lesions, failure to thrive, hypogonadism, impaired taste and smell as well as frequent infections due to a depressed immune system) [4].

We discuss a case of a two-year-old female infant with annular psoriasiform-eczematous skin lesions misdiagnosed as tinea corporis and psoriasis. Replacement therapy with oral zinc sulfate completely healed the skin lesions within two weeks of the onset of treatment.

## Case presentation

A two-year-old female, born of a standard spontaneous vaginal delivery with unremarkable prenatal, natal, and postnatal histories, presented with a history of slightly itchy, slowly progressing, and persistent skin lesions one month after weaning from breast milk at the age of five months. The patient had no history of diarrhea, hair loss, or irritability. She had been feeding well and had no history of poor diet intake. The parents stated that the child had been seen by many dermatologists and diagnosed with tinea corporis "despite a negative fungal culture" and treated with topical and systemic antifungals, including miconazole, terbinafine, and fluconazole, for several months without benefit. They also reported that the child had been diagnosed by some dermatologists with psoriasis "despite nonspecific skin biopsy findings mentioned in the histopathology report brought by the patient" and treated with topical corticosteroids-calcipotriene ointment for several months without improvement.

**Figure 1:**
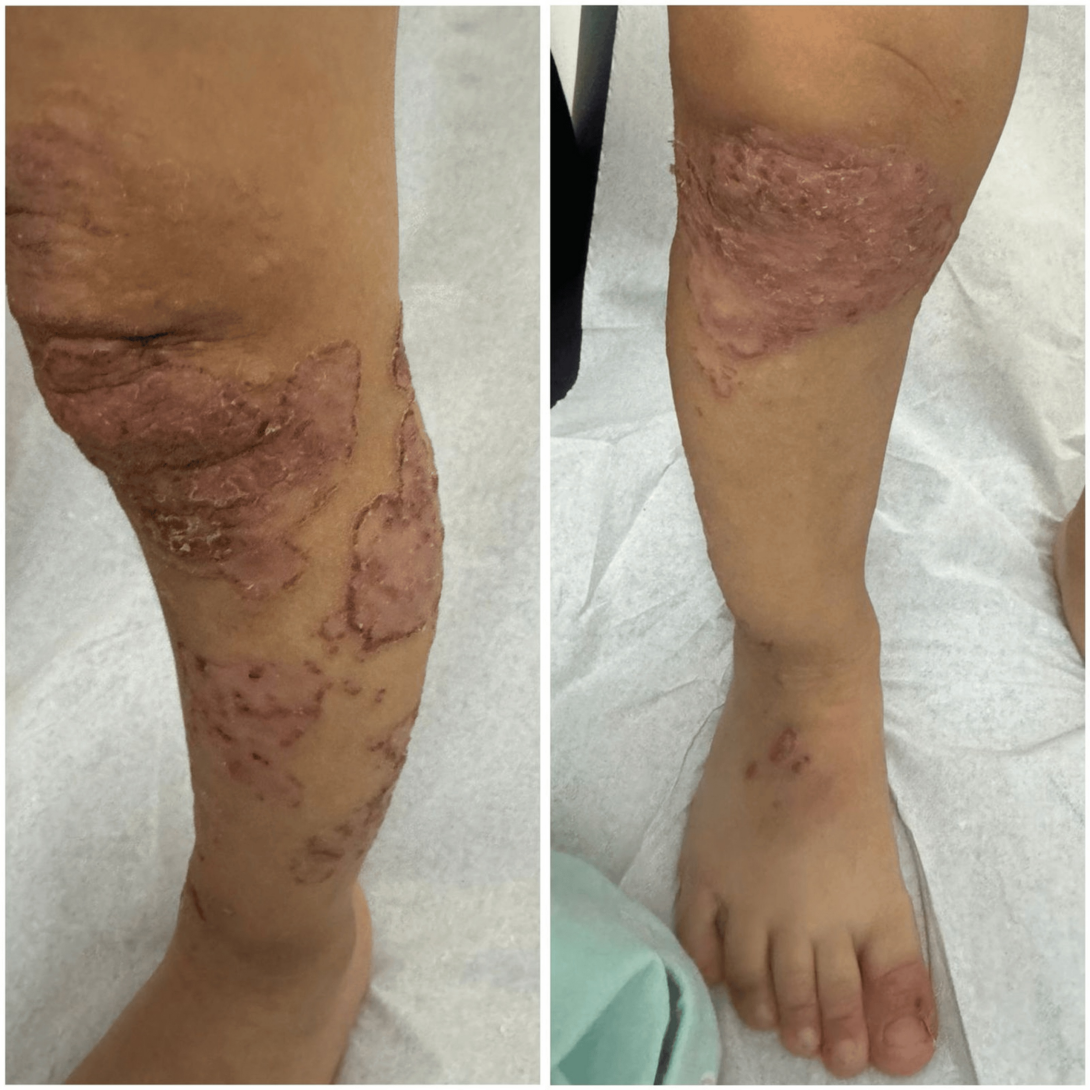
Multiple well-defined scaly erythematous plaques on the legs and feet, some with annular configuration

A review of systems, past medical history, and drug history were all unremarkable apart from the issues mentioned above. There were no similar cases in the family, and the parents were not consanguineous. A skin examination revealed multiple well-defined scaly erythematous plaques on all four extremities, and some lesions displayed an annular configuration (Figure 1).

**Figure 2:**
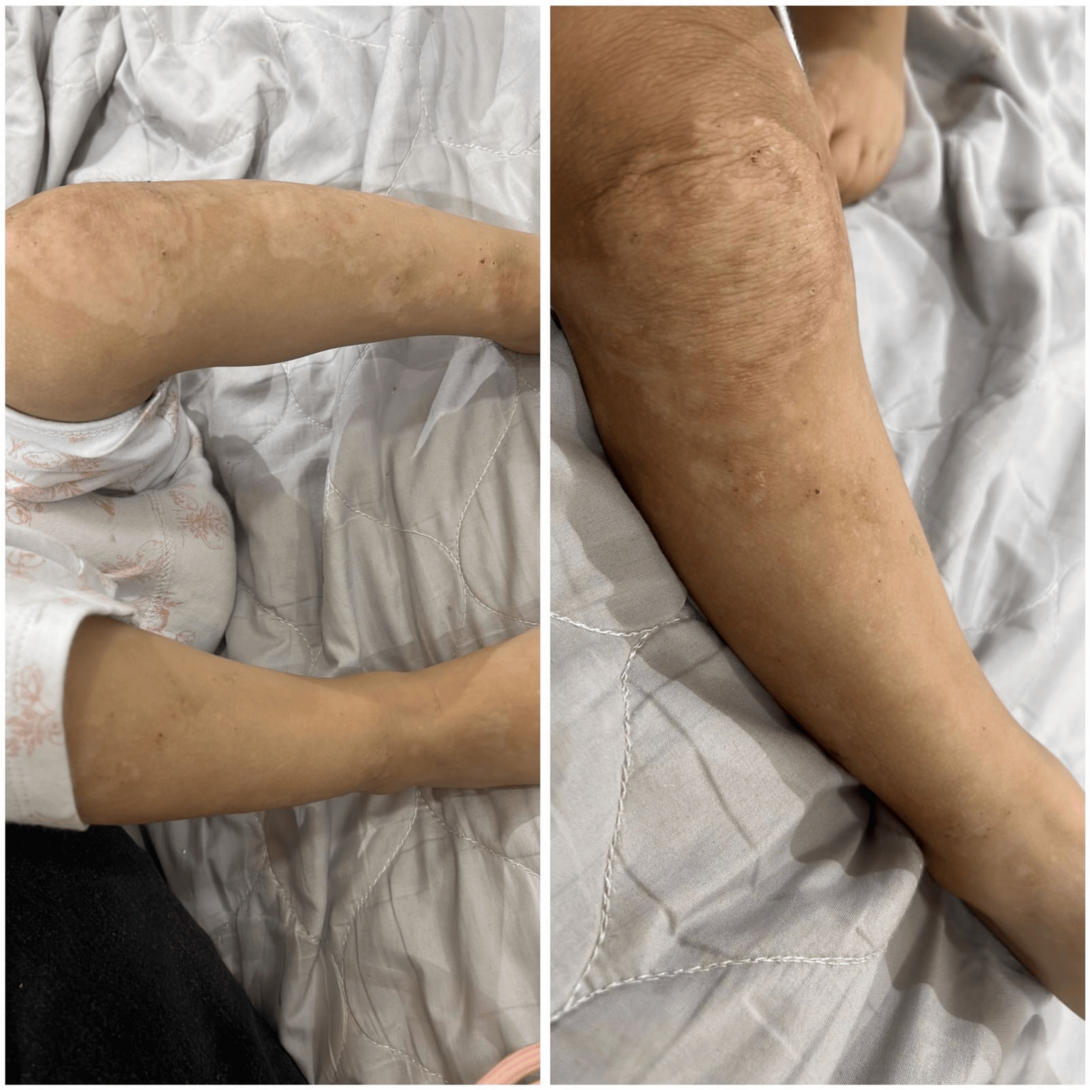
Post-inflammatory hypopigmented patches on lower extremities two weeks after initiating zinc therapy

The patient had mild angular cheilitis and very mild, diffuse, fine, scaly erythematous patches on the vulva. The differential diagnoses included tinea corporis, psoriasis, AE, and subacute cutaneous lupus erythematosus (SCLE). The skin biopsy from the patient was not repeated. The laboratory investigations revealed a low serum zinc level (49.10 ug/dL; normal range: 60-110 ug/dL). The complete blood count, urea, creatinine, urine examination, and liver functions, including alkaline phosphatase, were all within normal limits. Based on these clinical-laboratory findings, a provisional diagnosis of AE was made. A diagnostic test with zinc sulfate therapy was carried out. The patient was started on 13.2 mg/kg of zinc sulfate syrup, divided into two doses. After two weeks of zinc sulfate therapy, all skin lesions completely healed (Figure 2).

Based on a comprehensive assessment of all findings related to the patient, a final diagnosis of AE was made. The parents were advised to keep the patient on zinc treatment for life. The patient was scheduled for periodic follow-ups and was seen one year after the initiation of zinc treatment; her condition was found to be well-controlled, with no skin lesions.

## Discussion

AE, a hereditary autosomal recessive disorder, is caused by mutations in the SLC39A4 gene on chromosome 8q24.3, which encodes the zinc transporter protein ZIP4 that facilitates zinc absorption by the intestines [5]. AE is a rare disease and its incidence was estimated to be 1 in 500,000 births with an equal male-to-female ratio. Zinc is found in high concentrations in breast milk; thus, symptoms of AE, as observed in the patient, typically appear when infants are weaned from breast milk [6]. The differential diagnosis for annular scaly plaque in our patient included tinea corporis, psoriasis, SCLE, and AE [7].

The absence of a history of photosensitivity and the presence of nonspecific histopathological findings in the patient rule out the possibility of SCLE. The limitations of the diagnostic process in these cases are manifold, including the potential pitfalls of skin biopsy results, the low sensitivity and specificity of plasma zinc level, and the atypical clinical presentations. In the histopathology of the AE, the presence of pallor of the epidermis alone is nonspecific; however, its presence in combination with parakeratosis, epidermal atrophy, and vacuolization of the upper epidermis highly suggests nutritional deficiencies including zinc deficiency [4]. Nonetheless, these histopathological features were not mentioned in the histopathology report brought by the patient. Although the sensitivity and specificity of the plasma zinc level are low, it is a good indicator of zinc deficiency if there is a clear medical indication.

The low sensitivity can be attributed to the fact that the zinc level of the tissue is not correlated with that of the plasma. The low specificity is because a low plasma zinc level also occurs in several other conditions, including hypoalbuminemia, acute and chronic inflammatory conditions, and pregnancy [8]. Overall, a normal plasma zinc level in a patient with clinical features of AE does not rule out AE because the normal plasma zinc level could be due to blood-sample contamination by the tubes. Moreover, a high plasma zinc level can occur in catabolic states [8,9].

Thus, clinical correlation is crucial for an accurate diagnosis. In our patient, the atypical morphology of the skin lesions (annular configuration) as well as the lack of typical periorificial dermatitis decreased the threshold for a diagnosis of AE [9]. However, the presence of acral distribution of the skin lesions, fine scaly erythema on the vulva, angular cheilitis, and the onset of the skin lesions that had started after weaning were all good clues for the diagnosis of AE. Although low serum alkaline phosphatase supports the diagnosis of AE, it was normal in our patient. The low plasma zinc levels supported the diagnosis of AE in our patient. Ultimately, the diagnostic test with zinc sulfate therapy that led to the complete healing of all skin lesions within two weeks of zinc sulfate therapy confirmed the diagnosis of AE. Genetic testing for SLC39A4 mutations should be carried out in more challenging cases as it offers higher sensitivity and specificity.

In addition to genetic counseling for the parents, they were also informed about the requirement for lifelong treatment with zinc supplementation for the patient and the necessity of maintaining the least dosage for effective maintenance therapy with regular zinc level monitoring to avoid zinc toxicity.

## Conclusions

We described a case of a two-year-old female with widespread annular scaly plaques mimicking tinea corporis, annular psoriasis, and SCLE. No periorificial dermatitis was observed, which is atypical for AE. The absence of histopathological features of AE does not rule out a diagnosis of AE. The low serum zinc level in patients with atypical features of AE supports the diagnosis of AE. In such cases, a diagnostic test with oral zinc supplementation can confirm the diagnosis of AE if all skin lesions are healed within two weeks of the treatment. Therefore, a diagnostic test with oral zinc supplementation should be conducted in suspected cases of AE with normal serum zinc level or even if serum zinc level is low. Early diagnosis and prompt treatment of AE are critical to avoid complications that range from neuropsychiatric, ophthalmologic, and infection-related issues to death. Further research is needed to recognize and analyze more cases of this nature in regular practice.
